# Analysis of *Salmonella enterica* serovar Enteritidis isolates from chickens and chicken meat products in Malaysia using PFGE, and MLST

**DOI:** 10.1186/s12917-020-02605-y

**Published:** 2020-10-17

**Authors:** Zunita Zakaria, Latiffah Hassan, Zawiyah Sharif, Norazah Ahmad, Rohaya Mohd Ali, Suraya Amir Husin, Nor Hazrin binti Abd Hazis, Nor Fitriah Mohamed Sohaimi, Shafini Abu Bakar, Bashiru Garba

**Affiliations:** 1 Institute of Bioscience, Universiti Putra Malaysia, 43400 Serdang, Selangor Malaysia; 2 Bacteriology Laboratory, Department of Veterinary Pathology and Microbiology, Faculty of Veterinary Medicine, Universiti Putra Malaysia, 43400 Serdang, Selangor Malaysia; 3 Department of Veterinary Laboratory Diagnostics, Faculty of Veterinary Medicine, Universiti Putra Malaysia, 43400 Serdang, Selangor Malaysia; 4 Food Safety and Quality Division, Ministry of Health, 62675 Putrajaya, Malaysia; 5 Infectious Diseases Research Centre, Institute for Medical Research, National Institutes of Health, Setia Alam, Selangor Malaysia; 6 Diagnostic and Quality Assurance Division, Department of Veterinary Services, Ministry of Agriculture & Agro-Based Industry, Putrajaya, Malaysia; 7 Faculty of Veterinary Medicine, Usmanu Danfodiyo University, Sultan Abubakar Road, City Campus Complex, Sokoto, Sokoto State 840212 Nigeria

**Keywords:** *Salmonella* Enteritidis, Chicken meat products, Pulse field gel electrophoresis, Multi-locus sequence typing, Whole-genome sequencing, Antimicrobial resistance

## Abstract

**Background:**

*Salmonella* is a very important foodborne pathogen causing illness in humans. The emergence of drug-resistant strains also constitutes a serious worry to global health and livestock productivity. This study investigated *Salmonella* isolates from chicken and chicken meat products using the phenotypic antimicrobial screening as well as the molecular characteristics of *Salmonella* isolates. Upon serotyping of the isolates, the antimicrobial susceptibility profiling using a panel of 9 commonly used antimicrobials was done. Subsequently, the molecular profiles of all the isolates were further determined using Pulsed Field Gel Electrophoresis (PFGE) and the Whole Genome Multi-Locus Sequence Type (wgMLST) analysis in order to obtain the sequence types.

**Results:**

The PFGE data was input into FPQuest software, and the dendrogram generated was studied for possible genetic relatedness among the isolates. All the isolates were found to belong to the *Salmonella* Enteritidis serotype with notable resistance to tetracycline, gentamycin, streptomycin, and sulfadimidine. The *S*. Enteritidis isolates tested predominantly subtyped into the ST11 and ST1925, which was found to be a single cell variant of ST11. The STs were found to occur in chicken meats, foods, and live chicken cloacal swabs, which may indicate the persistence of the bacteria in multiple foci.

**Conclusion:**

The data demonstrate the presence of *S*. Enteritidis among chickens, indicating its preference and reservoir status for enteric *Salmonella* pathogens.

## Background

The continuous emergence of multidrug-resistant strains of non-typhoidal *Salmonella* constitutes a serious health hazard globally [1–4]. In recent years, *Salmonella enterica* associated with gastrointestinal infection in humans has been reported with increasing frequency worldwide [5]. *S.* Enteritidis is one of the most common causes of foodborne infection in humans [6]. While the majority of the infections are mild self-limiting illnesses, a small number have been reported to cause invasive infections, which is characterized by severe illness that requires hospitalization [7]. The popularity of *S.* Enteritidis is attributed to the unique ability of this serotype to contaminate chicken egg and meat without any discernible illness to the chickens [8]. Furthermore, multiple investigations have identified antimicrobial resistance phenotypes of *S. Enteritidis from among various food materials of poultry origin* [9]*.*

In Malaysia, retail chicken meat has been reported as a popular source of multiple antimicrobial-resistant *Salmonella* with *S.* Enteritidis accounting for 6.7% [10]. This multidrug-resistant (MDR) *Salmonella* are considered a serious public health problem due to tendencies for transmission of resistance to humans across the poultry production chain; thus it has become paramount to identify and characterize this important pathogen [11]. Moreover, concerns over the emergence of *Salmonella* with increased virulence, transmissibility, and antibiotic-resistance features, has necessitated the need for highly efficient methods that can identify these variant pathogens to track their spread especially across the human, animal and environmental interface [12]. In this regard, molecular techniques including whole genome sequencing, Pulse Field Gel Electrophoresis (PFGE), and Multi-Locus Sequence Typing (MLST) are among the commonly employed methods. These techniques can characterize pathogens in order to determine clonal and strain distribution across various environments and hosts.

Pulsed-field gel electrophoresis is one of the most widely used methods for the epidemiological studies of pathogenic bacterial organisms due to its high discriminatory ability [13]. With the globalization of trade, including poultry and poultry products, PFGE can be useful in understanding the diversity and evolution of infectious disease agents in order to evaluate their genetic relatedness to determine their point source during epidemiological investigations [14]. The principle of this method is based on the restriction enzyme digestion of whole DNA to produce fragment patterns that vary from strain to strain. The method relies on the distinct genomic differences between isolates that are observed as a result of the rapid accumulation of genetic variations that lead to slightly detectable differences between DNA fingerprints patterns within a clone [12].

Multi-Locus Sequence Typing (MLST) analysis, on the other hand, is best suited for longer-term as well as global epidemiologic investigations of infectious disease agents [12]. It is based on the principle of the multi-Locus enzyme electrophoresis that uses a combination of discriminatory power and clonal stability, which has proven to be extremely efficient in characterizing clones within a population of bacterial organisms causing serious disease [15]. However, it uses allelic differences in the sequence of various house-keeping genes that are often exploited to differentiate strains [16].

In this investigation, the phenotypic antimicrobial resistance profile and molecular characteristics of *S.* Enteritidis isolated from chicken and food isolates was assessed using Pulsed-field gel electrophoresis and Multi-Locus Sequence Typing (MLST) analysis in order to understand the molecular characteristics and the epidemiological distribution of the *S*. Enteritidis strains circulating in the Central region of Peninsular Malaysia from 2016 to 2018. This will help reveal the genetic relatedness and strain variabilities and distribution among the antimicrobial resistant isolates obtained from human clinical samples, live birds, and chicken meat in Malaysia.

## Results

### Serotyping of *Salmonella* and antimicrobial resistance

All of the 47 isolates were analyzed by the classical serotyping slide agglutination test comprising of ready-to-eat foods (7), chicken meats (11) and chicken cloacal swabs (29), in accordance with the White–Kauffmann–Le Minor scheme and only isolates belonging to the *S.* Enteritidis serotype were selected for this study. Based on the phenotypic antimicrobial resistance pattern of the isolates against the nine (9) different antimicrobial drugs, 27 (57.45%) of the isolates were found to show resistance to 1 or more antimicrobials tested (Table 1). However, out of the 20 that were susceptible to the drugs tested, 6 showed intermediate resistance to ampicillin, streptomycin, and tetracycline.

**Table 1:** Antimicrobial resistance pattern of the 47 *Salmonella* Enteritidis isolates

New ID	Source	AMR	ST	Remark
CM&MP01	Chicken meat	–	1925	Susceptible
CM&MP02	Chicken meat	TE	1925	Tetracycline resistant
CM&MP03	Food	–	11	Susceptible
CM&MP04	Food	TE	1925	Tetracycline resistant
CM&MP05	Food	–	11	Susceptible
CM&MP06	Food	TE	1925	Tetracycline resistant
CM&MP07	Food	TE	1925	Tetracycline resistant
CM&MP08	Chicken meat	–	11	Susceptible
CM&MP09	Chicken meat	TE	1925	Tetracycline resistant
CM&MP010	Chicken meat	TE	1925	Tetracycline resistant
CM&MP011	Food	TE	1925	Tetracycline resistant
CM&MP012	Food	TE	1925	Tetracycline resistant
CM&MP013	Chicken meat	–	1925	Susceptible
CM&MP014	Chicken meat	TE	1925	Tetracycline resistant
CM&MP015	Chicken meat	–	11	Susceptible
CM&MP016	Chicken meat	–	1925	Susceptible
CM&MP017	Chicken meat	–	1925	Susceptible
CM&MP018	Chicken meat	SXT	292	Sulfamethazine/Trimeth
CCS01	Chicken swab	TE	1925	Tetracycline resistant
CCS02	Chicken swab	TE	1925	Tetracycline resistant
CCS03	Chicken swab	–	1925	Susceptible
CCS04	Chicken swab	–	11	Susceptible
CCS05	Chicken swab	TE	11	Tetracycline resistant
CCS06	Chicken swab	–	11	Susceptible
CCS07	Chicken swab	AMP	11	Ampicillin resistant
CCS08	Chicken swab	AMP;CN;TE	11	**Multidrug resistant**
CCS09	Chicken swab	TE	1925	Tetracycline resistant
CCS010	Chicken swab	AMP	11	Ampicillin resistant
CCS011	Chicken swab	TE	1925	Tetracycline resistant
CCS012	Chicken swab	AMP	11	Ampicillin resistant
CCS013	Chicken swab	–	1925	Susceptible
CCS014	Chicken swab	–	1925	Susceptible
CCS015	Chicken swab	–	1925	Susceptible
CCS016	Chicken swab	–	329	Susceptible
CCS017	Chicken swab	–	1925	Susceptible
CCS018	Chicken swab	TE	1925	Tetracycline resistant
CCS019	Chicken swab	AMP	11	Ampicillin resistant
CCS020	Chicken swab	AMP	11	Ampicillin resistant
CCS021	Chicken swab	–	365	Susceptible
CCS022	Chicken swab	–	1925	Susceptible
CCS023	Chicken swab	–	1925	Susceptible
CCS024	Chicken swab	–	1925	Susceptible
CCS025	Chicken swab	TE;S;AMP	2132	**Multidrug resistant**
CCS026	Chicken swab	TE	1925	Tetracycline resistant
CCS027	Chicken swab	TE	1925	Tetracycline resistant
CCS028	Chicken swab	TE	1925	Tetracycline resistant
CCS029	Chicken swab	TE	1925	Tetracycline resistant

Key: CM&MP-Chicken Meat & Meat Products; CCS-Chicken Cloacal Swab

When stratified by the source of samples, *S.* Enteritidis isolated from food samples exhibited the highest number of resistance 5/7 (71.4%), followed by cloacal swabs 17/29 (58.6%) and then chicken meats with 5/11 (45.5%). However, chicken swab isolates had the overall highest resistance with 17/47 (36.2%). Importantly, only two isolates, both from cloacal swab samples, showed multi-drug resistance (CCS08 = AMP; CN; TE; CCS025 = TE, S, AMP). Moreover, the antimicrobial agent with the most resistance across all the isolates tested was tetracycline 21/47 (46.8%); While ampicillin had 7/47 (14.8%) and, streptomycin, sulfadimidine/trimethoprim, and gentamycin all had 1/47 (2.1%) respectively.

### Multi-locus sequence typing analysis

For the MLST of the completely sequenced bacterial genomes, short sequence reads were first assembled to draft genomes [17]. For the Whole Genome MLST scheme, the MLST allele of each locus was aligned to the genome using BLAST. After that, the ST was determined by a combination of the MLST alleles after close-matching of the selected alleles. The MLST typing of all the 47 isolates was based on the comparison of internal sequences of the *Salmonella* seven housekeeping gene fragments (*aroC, dnaN, hemD, hisD, purE, sucA,* and *thrA*). The 47 *S*. Enteritidis were subtyped into six (6) different STs, with the majority assigned to ST1925 (30) followed by ST11 (12), with ST292, ST365, ST329 and ST2132 assigned to only one isolate each. Worthy of note is the fact that ST1925 is a single locus variant of ST11. Additionally, while ST1925 and ST11 occurred in food materials, chicken meats, and cloacal swabs, ST292 was found only in the chicken while ST365, ST329 and ST2132 were all found in cloacal swabs.

### Pulse-field gel electrophoresis

**Fig. 1:**
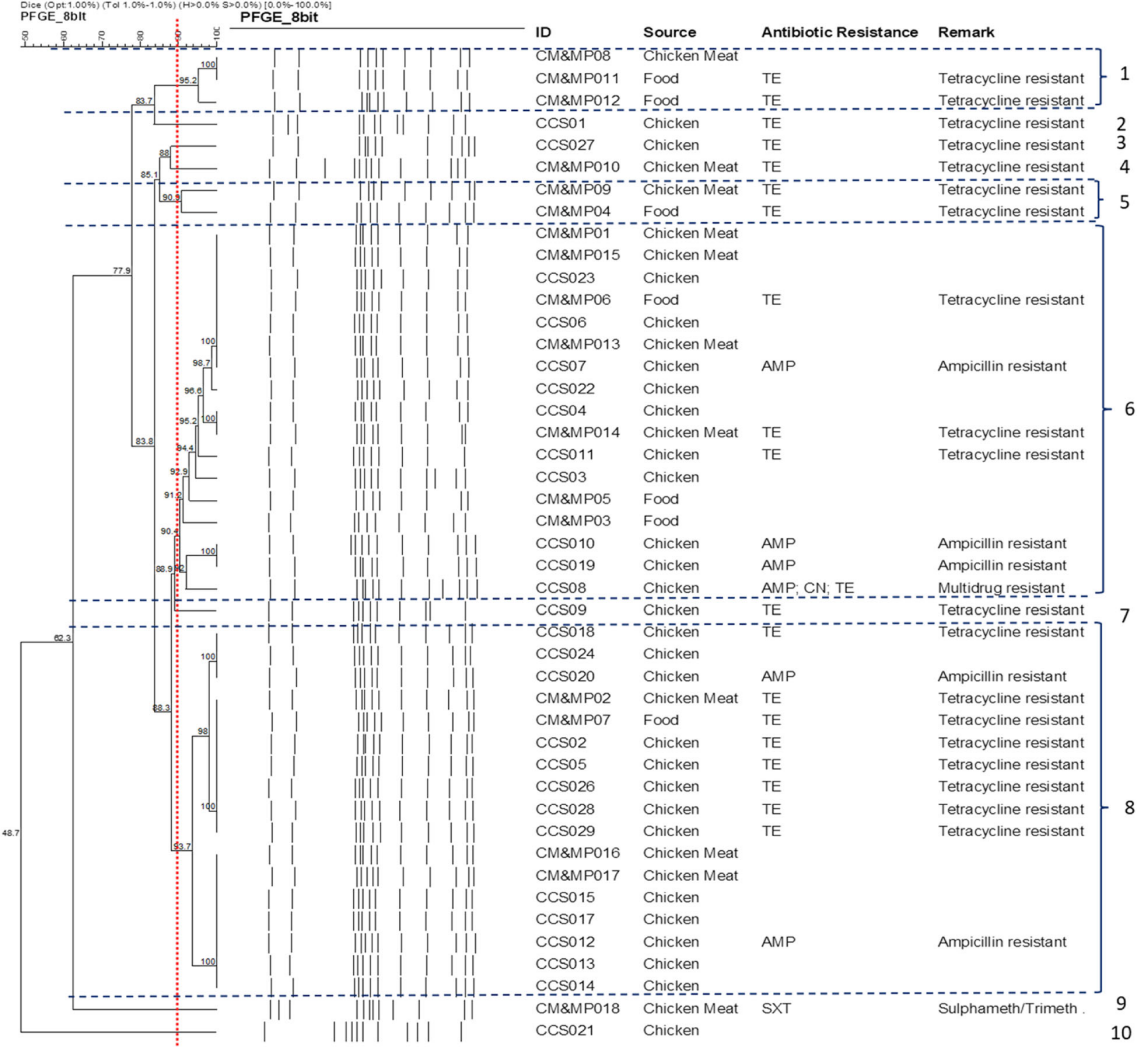
Pulsed-field gel electrophoresis (PFGE) patterns showing the DNA of the chicken meats, foods and chicken cloacal swab isolates digested by *Xba*I restriction enzyme (*n* = 45). Nine antimicrobial susceptibility patterns and 10 PFGE pulsotypes (1–10) were identified among the 45 isolates

The *Xba*I digestion was successfully performed on all the isolates except CCS016 and CCS025 (both from cloacal swabs), which were not typeable by PFGE, hence were excluded. However, the remaining 45 selected isolated yielded 9–13 DNA bands. With a Dice Coefficient of 0.5 and a similarity index of 90%, the PFGE analysis produced ten pulsotypes (1–10) with pulsotypes 6 and 8 being the major ones, pulsotype 1 and 5 had 3 and 2 isolates from chicken and meat isolates while 2, 3, 4, 9, and 10 appeared as singletons with 100% similarity. Moreover, the majority of the strains (17; 37.7%) belonged to pulsotype 6 and 8. Within the pulsotype 6, isolates from chicken meats, foods and cloacal swabs exhibited genetic relatedness ranging from 88.9 to 100%, likewise isolates in the pulsotype 8 shared a similarity score in the region of 92–100% (Fig. 1).

### Discriminatory ability

Simpson’s index of diversity (*D*) was used to compare the bacterial typing method based on MLST and PFGE pattern of the isolates. For the 10 PFGE types, *D* was 0.96 while for the six (6) sequence types identified by MLST, *D* was 0.99. These indices imply that if two isolates are to be sampled randomly from the population, then 96 and 99% of the time they will be assigned into different types. However, it is recommended that a good index should be greater than 0.95 [18, 19].

## Discussion

This investigation was undertaken to examine *Salmonella* isolates from foods sold at restaurants, chicken meats sold at supermarkets and wet night market in the central region of Peninsular Malaysia, as well as samples from live chickens from selected poultry farms located within the central region of Peninsular Malaysia in order to assess the antimicrobial susceptibility and the genetic relatedness of the *Salmonella* pathogen. In total, 47 *S.* Enteritidis were identified after the culture, isolation, biochemical characterization, and serotyping was done. In order to determine their genetic relatedness, whole-genome sequencing wgMLST and PFGE were conducted.

The antimicrobial susceptibility analysis of all isolates from the food sources, chicken meats, and chicken cloacal swabs exhibited susceptibility and varying resistance characteristics to the antimicrobial panel tested. As mentioned above, all of the 47 isolates were confirmed to be *S.* Enteritidis serotype upon slide agglutination test. The phenotypic antimicrobial resistance result showed that the majority of the isolates were resistant (57.45%) to the antimicrobials tested. Although only two isolates had multiple resistance (resistant to 3 or more), the majority of the isolates were resistant to tetracycline. The two *Salmonella* isolates with a multi-drug resistant profile were resistant to tetracycline, which has gained popularity as a clinically and agriculturally relevant antibiotic [20]. The National Pharmaceutical Regulatory Agency (NPRA), which is the drug control authority of Malaysia under the Ministry of Health (MOH), Malaysia and the Department of Veterinary Services (DVS) under the Ministry of Agriculture has granted approvals for the use of tetracycline in the treatment of disease, as prophylaxis and as growth promoters [21]. However, the inappropriate use of these antibiotics in the food-producing animals constitutes a serious public health hazard [22]. Moreover, the emergence of multiple antibiotic resistance, as observed in two isolates in this study, may progressively undermine the viability of many of the routinely used antibiotics.

Sequence analysis of the isolates found that the most common sequence type (ST) observed among all the isolates were ST1925 (30) followed by ST11 (12). While novel STs reported in Malaysia for the first time (ST292, ST365, ST329, and ST2132) were also detected in only one isolate for each. Comparison of our result against the MLST database indicates that ST1925 is relatively common among *S.* Enteritidis isolated from human and avian species from the United Kingdom, United States, Australia, and Malaysia (enterobase). While ST11 had been reported in humans, poultry, food, and some wild animal species, including reptiles in many countries from Asia, Africa, South America, and European countries (enterobase). Notably, ST1925 has been reported to be a single cell variant of ST11, and both sequence types are known to be geographically widespread and have previously been reported in foods, humans and animals [23, 24]. The detection of these STs from different sources may also indicate their ability to adapt to, and persist in different hosts or types of samples. The detection of ST11 in foods may imply possible transmission from neighboring countries like Singapore and China, where the ST type prevails [24]. In this study, the Whole-Genome Sequencing platform was used for the MLST analysis against the traditional PCR based MLST, followed by Sanger sequencing. This is because of its superior discriminatory power and efficiency in genetic detection of variability between isolates, in addition to the fact that the traditional method is both costly and time-consuming [17]. Furthermore, ST292, ST365, and ST2132 have been previously reported in Singapore (aquatic), India (human & environmental samples), and United States (poultry) while ST329 was reported among isolates obtained from poultry feed in Peru [25–28]. In this study, ST292 was detected in chicken meat, while ST365, ST329 and ST2132 were detected in the chicken cloacal swabs. When analyzed in the MLST database, these strains were not as common as the other STs detected except for ST365, and ST2135 which were found in poultry in the US from *Salmonella* isolates belonging to the Kentucky serotype. In addition, ST292 and ST2132 showed resistance to sulfadimidine/trimethoprim as well as streptomycin and tetracycline respectively. Therefore, having demonstrated similar antimicrobial susceptibility profiles as well as a common source, this may be suggestive of possible strain relatedness, and potential for transmission between chicken and ready-to-eat foods.

The pulsed-field gel (PFGE) analysis of the isolates revealed that most of the *S.* Enteritidis isolates examined exhibited unique genetic relatedness, albeit with some variability. Cluster analysis identified ten (10) pulsotypes with the majority belonging to pulsotype 6 and 8, which further had 17 subtypes that shared 100% identity pattern between the poultry and food source. From the results of the PFGE analysis, it was evident that PFGE revealed more significant differentiation (10 profiles) compared to the MLST, which produced six (6) sequence types. The earlier observation supports this finding that the diversity indices with PFGE produced the highest rate of variability over MLST and the phenotypic antimicrobial susceptibility testing [29]. On the other hand, a one-to-one correlation between PFGE types and ST revealed that some isolates belonging to the same PFGE type had multiple STs and vice versa. The result of this study showed that both MLST and PFGE had a high index of discrimination (*D =* 0.95) above the recommended value. The slight disparity with respect to the ‘Simpson’s index between MLST and PFGE observed in this study has previously been reported where MLST was found to exhibit higher discriminatory power with respect to the typing of Extended Spectrum Beta Lactamase *Escherichia coli* [19]. The authors argued that such disparity might be attributed to the spectrum of changes detected by PFGE and MLST. In other words, while PFGE detects changes in nucleotide sequence associated with insertions or deletions of DNA, MLST typing detects nucleotide changes within an amplified gene fragment [19]. However, in recent years, the potentials of molecular techniques in discriminating between strains of *S*. Enteritidis have become more pronounced. Methods with the highest discriminatory power are more specific and therefore, better recommended during investigations of closely related isolates [30]. Furthermore, the PFGE analysis delineated the genetic variability between the *S*. Enteritidis isolates from a different source based on the distinct DNA fingerprints generated. Except for two isolates, all other isolates were type-able and the technique reproducible, which could be very useful as an epidemiological tool for disease outbreak investigation. The present study also showed that various PFGE subtypes identified are present in both fresh chicken meat, live birds, and even cooked food ready for eating. Although the DNA profiles of most of the *S.* Enteritidis isolates from various sources differed, which may indicate that the isolates belong to different clones as revealed by the MLST analysis.

## Conclusion

This investigation has highlighted the usefulness of molecular and phenotype analysis in understanding the genotypic and phenotypic characteristics of *S*. Enteritidis. A higher level of diversity was observed among the *S*. Enteritidis isolates based on the PFGE, which is an indication of its potential as the molecular choice technique for the subtyping of isolates of the same serovar. The five sequence types detected in the present study with the wgMLST analysis also showed host variability by occurring in live chickens, cooked foods, and fresh chicken carcasses in addition to the other animal and human hosts upon comparison with the MLST database. The antimicrobial resistance pattern was equally evident in food isolates as compared to the other sources investigated in this study.

## Methods

### *Salmonella* isolates

All the *Salmonella* isolates used in this study were obtained from the laboratory collection of the Food Safety Division, Ministry of Health Malaysia (7), and the Department of Veterinary Services Malaysia (40). After the differential culture, isolation and biochemical characterization, all the isolates were suspended in a Brain Heart Infusion (Oxoid) broth supplemented with 20% glycerol and then stored at − 80 °C until required. All the isolates (47) comprised of 29 cloacal swab, seven ready –to-eat foods and 11 fresh chicken portions of meat at retail outlets.

### Serotyping and antimicrobial susceptibility testing

The *Salmonella* serotype (*S.* Enteritidis) was determined using the slide agglutination assay according to the Kauffman-White scheme based on the agglutination of the bacteria with commercial *Salmonella* O (somatic) and H (flagellar) antisera (DIFCO, Detroit, Mich., USA) in order to identify variants of the O and H antigens. While the antimicrobial susceptibility tests were conducted using the disk diffusion method per the Clinical Laboratory Standard Institute (CLSI) protocol [31]. The isolates were tested against ampicillin (Amp), chloramphenicol (C), gentamicin (CN), streptomycin (S), sulfamethazine/trimethoprim (SXT), tetracycline (TE), ceftiofur (EFT), cefotaxime (CTX), and ciprofloxacin (CIP).

### Whole-genome sequencing and MLST

The QIAamp DNA Mini Kit (Qiagen, Hilden, Germany) was used to extract and purify the genomic DNA from all the *S.* Enteritidis isolates. While the NGS library preparation was achieved with the aid of the Nextera XT DNA Sequencing Library Preparation Kit (FC-131-1096; Illumina, San Diego, California, USA) following the manufacturer’s instructions, and the sequencing was done using the Illumina NextSeq sequencer by scanning for adapter sequences followed by removal of low-quality sequences. Finally, sound quality sequencing reads were assembled de novo using SPAdes software version 3.9.0 (BioEasy Sdn Bhd). The Whole Genome Sequences generated were analyzed with the aid of the software EPInod (BioEasy Sdn. Bhd. Malaysia). Multi-locus Sequence Typing was conducted within the EPInod suite by streamlining all the sequences for the isolates on the MLST program against the PubMLST database (MLST version 2.6).

### PFGE analysis

PFGE analysis was performed based on the standardized protocol for the subtyping of *Salmonella* in the PulseNet [32]. Purified DNA was digested using *Xba*I restriction enzyme (NEB) in a final volume of 100 μl and incubated at 37 °C for 3 h and embedded in a 1% SeaKem Gold Agarose (Sigma Aldrich) prepared using 0.5x TBE buffer. The reaction was run for 18 h using the Chef Mapper XA system (Bio-Rad) in order to resolve the DNA macro-restriction fragments. *Salmonella enterica* Typhimurium was used as a control. Macro-restriction patterns were compared using the FPQuest cluster analysis based on the Dice correlation coefficient, while dendograms were constructed using the unweighted-pair group method using average linkages UPGMA.

### Data analysis

*Salmonella* isolates were assigned sequence type (ST) according to their allelic profiles corresponding to the seven housekeeping genes, while the PFGE patterns were expressed as pulsotypes. Simpson’s index of diversity (*D*), which measures the index of discrimination for the two typing methods was calculated using the formula below [18]:

$$ D=1-\frac{1}{N\left(N-1\right)}\sum \limits_{j=1}^S{n}_j\left({n}_j-1\right) $$

According to the formula, *N* is the total number of strains in the sample population, *S* is the total number of types described, and *n*_*j*_ is the number of strains belonging to the *j*th type.

## Data Availability

The datasets analyzed during the current study are available from the corresponding author on reasonable request.

## Abbreviations

PFGE: Pulsed Field Gel Electrophoresis
wgMLST: Whole Genome Multi-Locus Sequence Type
MDR: Multidrug-resistant
MLST: Multi-locus Sequence Typing
DNA: Deoxyribonucleic acid
MOH: Ministry of Health
DVS: Department of Veterinary Services
ST: Sequence Types
